# Who Are Our Students? Understanding Students' Personality for Refined and Targeted Physical Education. A Scoping Review

**DOI:** 10.3389/fspor.2019.00031

**Published:** 2019-09-20

**Authors:** Alina Kirch, Melina Schnitzius, Filip Mess, Sarah Spengler

**Affiliations:** Department of Sport and Health Sciences, Technical University of Munich, Munich, Germany

**Keywords:** personality, students, physical education, school sports, teaching, scoping review

## Abstract

Students' personality is an essential component in order to plan and teach physical education (PE) lessons according to students' individual needs. Additionally, personality formation in general is part of the educational mandate and student personality development specifically is considered as an elementary goal of PE. Although student personality is a central topic in the PE context, the state of research, especially regarding the underlying personality understandings, is diverse and hard to capture. Therefore, this scoping review aims to (I) describe the underlying personality understandings and (II) analyze research questions and results of studies examining students' personality in PE. We conducted a scoping review. Eleven databases were chosen because of their specification within the field of education, sports and health sciences. We included references if they empirically examined students' personality in PE and were published in German or English. Twenty-four studies were included in the review. Fifteen of the included studies were cross-sectional, nine longitudinal. Regarding aim I), the underlying personality understandings were inconsistent across the studies but most of the studies followed trait theory. Considering aim II), the included studies investigated relationships between students' personality and either (a) students' achievement in PE, (b) students' psychological determinants of PE participation (e.g., motivation, anxiety), or (c) a school sports intervention. Results indicated that e.g., extraverted students tend to enjoy PE more and obtain less anxiety in PE. The review showed that students' personality in PE is empirically examined but the studies' underlying personality understandings, research questions and results are diverse. Findings highlight that PE contributes to students' personality development. Additionally, the review showed that results of personality research in PE context can be used in order to teach PE in a student-centered way (e.g., by deducing the detected relationships considering *extraversion*) and by this support students' lifelong physical activity. Further and targeted research in this field can help PE teachers to tailor their teaching to their students' needs. This increases the chances to achieve PE's two main goals—“*educating to sports (e.g., personality-aligned lessons addressing different motives)”* and “*educating through sports (e.g., personality development)”* in the long term.

## Introduction

Physical education (PE) fulfills an outstanding role within the school curriculum. PE is the only subject in which children are physically active (Penney and Jess, 2004). Even more distinguishing is the fact that PE is the only context in which all school-aged children experience instructed physical activities in the course of their lives (Tammelin et al., 2016). PE is therefore the only sure opportunity to get everyone on the move and convey the importance and chances of physical activity for a healthy life (Kohl and Cook, 2013). This opportunity and the associated goal in its core is internationally prevalent in PE's central assignment (Sallis and McKenzie, 1991; Pühse and Gerber, 2005; Scheid and Prohl, 2012). In Germany, PE's central assignment is typically characterized by two main goals (Scheid and Prohl, 2012): (1) Prepare and motivate students for a physically active lifestyle. In this regard, children need to explore different kinds of sports, acquire an appropriate range of movement skills and by this find their individual motives to be physically active in and outside school. PE supports discovering the personal meaning of physical activity and at the same time promotes the understanding and knowledge of various aspects of movement. Students by this develop the capacity to act on one's own and apply these competencies to a purposeful use of their leisure time and ideally lifelong physical activeness. PE has evolved to become a content area with diverse aims that facilitate the holistic—physical, social, emotional, and intellectual—development of children (NASPE, 2004). Part (2) of PE's central assignment therefore includes the goal of empowering students' personal development. In this regard, curricula claim that PE contributes to children's development in different facets, such as formatting and developing positive personal, social or emotional qualities.

### The Importance of Students' Personality in School

Students are in the focus of both abovementioned goals. PE's allocated educational mandate therefore implies the necessity to consider the learner in teaching processes such as lesson planning and implementation. In the general educational context, learners' individual needs are a central factor regarding their learning processes (Jurik et al., 2015). Knowing learners' individual needs in order to adapt teaching processes includes knowing the learners' personality. Personality formation is a central factor of the educational mandate which accounts for considering students' personality in teaching and research. Personality research in school showed a pervasive influence of personality traits on student outcomes such as students' well-being, emotional states or academic performance (O'Connor and Paunonen, 2007; Poropat, 2009; Richardson et al., 2012). According to O'Connor and Paunonen (2007) students' personality traits (Big Five) predict their academic performance in two different ways: (1) Via behavioral tendencies affecting habits (Rothstein et al., 1994) and (2) via students' willingness to perform (Furnham and Chamorro-Premuzic, 2004). O'Connor and Paunonen (2007) results further indicated the increasing importance of personality traits' influence compared to cognitive abilities' influence on academic performance when students become older (Furnham et al., 2003). In summary, students' personality plays a significant role in shaping their educational experiences (Matthews et al., 2006).

### Understanding Personality

In order to examine the importance and impact of students' personality in particular contexts, it is essential to conceive the underlying understandings of personality. Personality is a broad term describing a multifaceted construct (Johnson and Christensen, 2017). General personality research differentiates between seven major approaches of personality psychology: Psychoanalytic (i.e., Freud, 1940), neo-psychoanalytic (i.e., Adler, 1930; Jung, 1958), humanistic (i.e., Maslow, 1970; Rogers, 1972) emphasizing a self-actualizing tendency, behavioral (i.e., Watson, 1930; Skinner, 1972), biological (i.e., Sheldon, 1963; Cloninger, 1999), cognitive (i.e., Bandura and Walters, 1963; Ellis et al., 2009), and trait psychological (i.e., Cattell, 1946; Eysenck and Eysenck, 1969). In general personality research the trait approach became prevalent over time. Personality is therefore often defined as a person's unique structure of relatively stable traits (Guilford, 1971).

In order to interpret and compare the results of different studies following personality's trait theory, it is essential to know the different trait models' origin, their development and individual composition. In the course of time, some models have significantly influenced the development process of trait theory in general. Even if the models' origin varies, the chosen dimensions mostly display great relationships (Gerbing and Tuley, 1991; Goldberg and Rosolack, 1994). Initial trait psychological models are based on a lexical approach describing personality in multiple adjectives. Cattell (1946) derived 16 source traits inherent in every person. Cattell's 16 primary factors are categorized in 5 s stratum source traits (Cattell, 1956; Rossier et al., 2004). The dimensions warmth, liveliness, social boldness, privateness, and self-reliance are summarized in the factor extraversion. The dimensions emotional stability, vigilance, apprehension, and tension are subordinate to anxiety. Tough-mindedness is a combination of warmth, sensitivity, abstractedness and openness to change. Independence unites the dimensions dominance, social boldness, vigilance, and openness to change. Self-control includes the dimensions liveliness, rule-consciousness, abstractedness, and perfectionism (Cattell, 1956; Rossier et al., 2004). Cattell's (1946) model became a standard personality measure in about 1970. At about the same time, Eysenck and Eysenck's (1969) model which, contrary to Cattell's, describes personality in broad, abstract terms, was developed. Eysenck focused more on biological traits and revealed two major dimensions: Introversion vs. extraversion and emotional stability vs. emotional instability. Later he added the third dimension psychoticism (Eysenck, 1976). Eysenck (1984) stated that his and Cattell's (1946) model should not be seen as contradictory but rather as complementary and mutually supportive. An analysis estimating the two models' comparability confirmed the equivalence of the factors anxiety and neuroticism as well as the equivalence of the models' extraversion factors (McKenzie, 1988). At the end of the 20th century, McCrae and Costa (1987) as well as Goldberg (1990) developed two similar models, which differed mainly in their mode of formation. While Goldberg (1990) pursued a lexical approach and developed the model of the Big 5, McCrae and Costa (1987) empirically analyzed personality questionnaires and by the means of factor analysis developed their five-factor model. Both models unite roughly the same five personality dimensions: Openness to experience, conscientiousness, extraversion, agreeableness and neuroticism. The five-factor model is currently the most prevalent model in personality research in general in order to describe personality holistically and superseded the aforementioned models (Cattell, 1946; Eysenck and Eysenck, 1969). The similarity between Cattell's global scales and the five-factor model was confirmed too. The two models share four of the five global dimensions. Only the dimension agreeableness is not represented in Cattell's 16 PF (Rossier et al., 2004). Due to the content-related similarity between different trait models, results of studies based on these models are to a certain extend comparable.

### Considering Students' Personality in PE

This knowledge on personality research's development and its current orientation is essential for further investigating students' personality specifically in PE context. As previously mentioned, numerous relationships between students' personality and learning outcomes have been ascertained in the general educational context. It seems logical that the detected relationships—examined on this general level—also exist on a more specific level, e.g., considering PE particularly. Due to the fact that the PE context particularly creates incentives and opportunities contributing to students' personality development (Kohl and Cook, 2013), examining relationships between students' personality and learning outcomes in PE becomes important. Even though research has demonstrated that students' personality is related to various factors influencing academic performance (Komarraju et al., 2011) and PE's allocated mandate postulates students' personality development (Scheid and Prohl, 2012), research considering students' personality in PE has been very rare so far. Most studies investigate only single aspects related to personality, such as students' attitudes toward or perceptions of PE (e.g., Harwood et al., 2015; Kretschmann, 2015; Silverman, 2017). In order to capture the complex construct of personality and by this its impact on students' physical activity and their personality development, it is insufficient to only describe individual components of students' personality (Asendorpf and Teubel, 2009). Asendorpf and Teubel (2009) therefore claim to examine students' personality following an integrative perspective within the context of a holistic personality development. This fosters a better understanding of PE specific outcomes such as students' motor performance, achievement motivation and the development of personal, social, or emotional competencies. Holistically understanding students' personality allows to identify and address students' needs—part one of PE's main goals (“educating to sports”)—and provides links for students' personality development—part two of PE's main goals (“educating through sports”) (Sallis and McKenzie, 1991; Siedentop, 2009; Scheid and Prohl, 2012).

A review article of existing literature examining students' personality in PE would be beneficial to summarize findings and by this ideally highlight the potential of personality research in the PE context. Due to diverse personality understandings, different research questions and investigated correlates within studies, the identification of relevant literature is challenging. Hence, a broad approach is essential in order to capture all relevant texts. A review of this kind—considering students' personality in PE following a wide approach to provide an overview of the existing literature on a general level—does not yet exist. Review articles in PE context are mostly concerned with PE teachers—often focusing on their education (Scheuer, 2019) or teaching methods (Lander et al., 2017). Review articles, considering the students in PE, typically focus on specific questions concerning students' personal characteristics and within this individual aspects such as self-concept or achievement motivation, rather than the students' personality in a broad sense (Kretschmann, 2015; Ang and Yubing, 2017; Silverman, 2017). The latter approach is more common in general educational research. Here studies conclude with promising results, e.g., detecting a relationship between students' personality and academic performance (O'Connor and Paunonen, 2007).

In the specific field of PE, reviewing the literature considering students' personality following a broad approach has not been conducted so far. Therefore, the aim of our review was to provide an overview of studies proclaiming to assess students' personality in PE. More precisely, we intended to (I) describe the underlying personality understandings by analyzing the pursued personality approach and applied personality inventories and (II) depict the studies' research questions and associated results by analyzing investigated variables, relationships or outcomes.

## Methods

Scoping reviews are especially helpful in order to provide a broad picture of existing literature in a wide research field (Booth et al., 2016), such as personality research. Due to the fact that personality research is carried out in various contexts and due to the existence of diverse personality understandings, we decided to conduct a scoping review.

### Selection Criteria

We were interested in investigating the students' *(sample)* needs, more specifically their general requirements regarding their personality *(content)*. Further, we were specifically interested in studies examining these needs in PE *(context)*. Therefore, we had to predefine our inclusion criteria, which also formed the basis of the search term in the three following categories: (1) Study focused on personality or rather proclaimed to assess personality; (2) sample under consideration comprised primary or secondary school students; (3) study was carried out during PE lessons or in school sports contexts. Category (1) was searched on title-abstract-keyword level in order to make sure that personality was the key issue in the text. The reference had to focus on personality or at least mention it as variable or outcome. Category (2) and (3) were searched on full-text level and included synonyms for students and various school sports contexts. Additionally, the publication language had to be English or German. The publication period was not limited and all publication types were considered, which is in line with Arksey and O'Malley's (2005) methodological guidelines of scoping studies.

### Search and Review Process

Based on Arksey and O'Malley's (2005) recommendations, the search strategy comprises four sequential steps: (1) Initial electronic database search; (2) key journal search of the included studies; (3) reference list search of the included studies; (4) manual author search of authors of the included studies. Considering the aforementioned principles the following search terms were used in the database search (in the following exemplary for the database Scopus): (TITLE-ABS-KEY ((persönlichkeit^*^ OR personalit^*^ OR schülerpersönlichkeit^*^)) AND ALL ((schüler^*^ OR kinde^*^ OR jugend^*^ OR student^*^ OR pupil^*^ OR schoolchild^*^ OR scholar^*^ OR kid^*^ OR child^*^ OR youth^*^ OR learner^*^ OR adolescen^*^ OR teen^*^ OR youngster^*^)) AND ALL ((sportunterricht^*^ OR schulsport^*^ OR bewegungserziehung^*^ OR bewegungsunterricht^*^ OR leibeserziehung^*^ OR leibesübung^*^ OR “physical education” OR “gym^*^ class^*^” OR “school sport^*^” OR “physical training”))) AND (LIMIT-TO (LANGUAGE, “English”) OR LIMIT-TO (LANGUAGE, “German”)). In total, 11 databases were searched: Education Source, ERIC, PsychARTICLES, PsycINFO, PSYNDEX, PubMed, Scopus, SocINDEX, SPOLIT, SportDiscus and Web of Science. The databases were chosen because of their specification in the field of education, sports and health sciences. The first search was realized on February 6th 2017. An update search to ensure the review's topicality was implemented on June 27th 2018. The first and second author functioned as two independent reviewers and fulfilled the screening process—first on title and afterwards on abstract level, independently deciding whether the reference should be included or not. References were excluded if not published in English or German, not empirical, not examining students, not in school setting and not investigating personality. In case of uncertainty (e.g., missing information, unsure about sample) the references were reassessed in the next step. Conflicts were discussed and solved collaboratively. We did not exclude studies due to quality reasons in order to examine the whole body of literature and by this be in line with the scoping review's methodological standards (Arksey and O'Malley, 2005). Last, full-texts were screened considering the same criteria as mentioned above.

**Figure 1:**
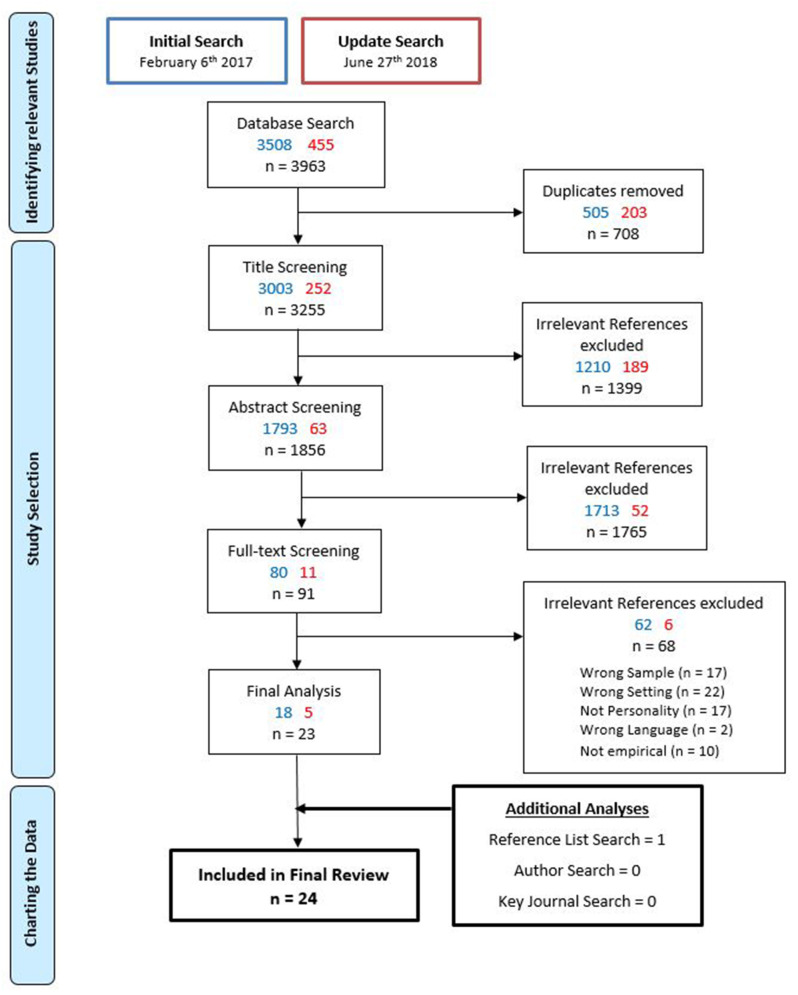
Flow chart of the screening and reference selection process.

The journals *Research Quarterly* and *sportunterricht* frequently appeared as publication source of the included studies. The manual key journal search was therefore applied to these two journals. Furthermore, the reference lists of all included studies were screened and all therein potentially relevant references had to pass the aforementioned screening process. As a final step, the included studies authors' research activities were investigated by entering the authors' names in the abovementioned databases and additionally checking their profiles and publication lists. If relevant research on students' personality in school sports contexts was detected, the publications were considered for potential inclusion and again had to run the screening process. Figure 1 summarizes the search and review process.

### Data Extraction and Analysis

In order to guide the data extraction stage, a data charting form in table format was created. The two reviewers first independently extracted the relevant information and filled in the table. Second, the two reviewers compared and discussed their tables, removed conflicts and joined the two tables to the final table. Subsequently, the variables under investigation in the included studies were extracted and grouped thematically. Further, results within these thematically similar groups were compared (within group comparison). For this purpose, the reviewers examined the possible comparability of the different applied inventories within a group. If a comparison was possible, e.g., due to a similar personality understanding pursued in the studies under investigation, the reviewers checked for replicability of the individually examined relationships among the studies.

Following Richards et al. (2017) as well as Arksey and O'Malley (2005) the results of the abovementioned data extraction and analysis step are presented in two formats. First, the results are summarized in table format (Table 1). Table 1 presents the pertinent information of the included studies. In addition to each study's framework conditions, the table includes the study's aim, underlying personality understanding (approach and applied inventory) and main results. Table 1 therefore provides a clear and compact presentation of answers to the review's research questions. Second, the results are provided in the running text, divided into framework conditions (author, year, origin, publication type, and sample) and a thematic analysis which explicitly addresses the review's research questions and provides an elaborated analysis.

**Table 1:** Included studies' framework conditions, aim, personality understanding, and main results.

**Framework conditions**		**Aim**	**Personality understanding**		**Main results**
**Author (Year) Origin—Publication Type: *Journal Name***	**Sample**		**Personality approach**	**Personality inventory**	
**CROSS-SECTIONAL STUDIES**					
Culjak and Mlačić (2014) Croatia—Journal Article: *Croatian Journal of Education*	100 students (59 m; 41 f); grade 1 and 2 high school (age 16–17)	Relationships between personality and success (good grade) in PE	Big five model of personality (Goldberg, 1990)	Questionnaire: IPIP100 (Mlacic and Goldberg, 2007)	- Personality is related to success in PE - Students' success was positively related to conscientiousness and emotional stability (in girls) and negatively to extraversion (in boys)
Dunkerbeck and Prenner (1976) Germany—Book Section	50 PE teachers	Proof and analysis of implicit personality theories in PE context	Implicit personality theories; stereotypes	1)Free description of “the underperforming student” 2)Characterization within given dimensions	Implicit theories of PE teachers contain four dimensions to describe personality of students: physical abilities and conditions; PE expectations; sociability and interactive recognition; behavior
Erpic et al. (2005) Slovenia—Journal Article: *International Journal of Physical Education*	1,025 students; grade 5 and 7 primary school & 1 and 3 secondary school (age 11–18)	Relationships between students' personality traits and(a) attitudes toward PE and (b) motivation for PE	Big five model of personality (Goldberg, 1990), ATEAQ (Erpic et al., 2005)	Questionnaire: B5P-C (Little and Wanner, 1998)	a)Students scoring higher in conscientiousness show more positive attitudes toward PE b)Students scoring lower in agreeableness and higher in neuroticism are less motivated in PE
Friedrich (1978) Germany—Journal Article: *International journal of physical education*	523 students (257 m; 266 f); high school (age Ø 12.5)	Relationships between personality and achievement (good grade) in PE	Two-factor model (Eysenck and Eysenck, 1969)	Questionnaire: Hanes KJ (Buggle and Baumgärtel, 1975)	Students scoring higher in extraversion show better PE grades
Guszkowska and Rychta (2007) Poland—Journal Article: *Human Movement*	455 students (213 m; 242 f); high school (age 15–17)	Relationships between personality and students'(a) physical fitness and (b) gender-related diversification	Five-factor model (McCrae and Costa, 1987) 16 PF (Cattell, 1946)	Questionnaires: FCB-TI (Zawadzki and Strelau, 1997); polish version of NEO-FFI (Zawadzki, 1998); polish version of HSPQ (Rychta and Guszkowska, 2000)	a)Personality traits are poorly correlated with the adolescents' physical fitness b)Predictors of physical fitness are different in boys and girls. In boys: extraversion is positively correlated with the total fitness score, agreeableness is correlated with agility, trunk muscle strength and suppleness; trunk muscle strength and suppleness also with conscientiousness. None of these correlations are shown in girls
Hayes (2017) UK—Journal Article: *Research Papers in Education*	296 students (150 m; 146 f); primary school (age 5–11)	Analysis of factors responsible for negative attitudes toward PE	Personality part of “Personal factors” (variable + intrinsic)	Semi-structured interview	Identified factors: lack of self-efficacy, a lack of perceived autonomy, family and peer factors and individual physical and personality factors are decisive for negative attitudes toward PE
Kerr (1978) UK—Journal Article: British *Journal of Physical Education*	165 students (97 m; 68 f); grammar school (age 11–12)	Relationships between personality variables and physical ball skills	Personality = mind and body (physical, intellectual, social and emotional) 16 PF (Cattell, 1946) Two-factor model (Eysenck and Eysenck, 1969)	Questionnaires: JEPI (Eysenck, 1965); HSPQ (Hundleby and Cattell, 1968)	Students with good physical ball skills score higher in warmth, emotional stability, dominance, liveliness and extraversion and score lower in sensitivity, social boldness and apprehension or introversion
Klein (2017) Germany—Journal Article: *sports*	1,399 students (707 m; 692 f); grade 7 (age Ø 12.9) and 10 (age Ø 15.8)	Relationships between physical self-concept and general personality traits	Big five model of personality (Goldberg, 1990)	Questionnaire: NEO-FFI (McCrae and Costa, 1992)	Students scoring higher in neuroticism assess their own physical attractiveness and own athleticism lower
Lodewyk and Gao (2018) USA/Canada—Journal Article: *International Journal of Sport and Exercise Psychology*	319 students (162 m; 157 f); grade 9 and 10 high school	Relationships between personality traits and(a) enjoyment and (b) effort in PE as a function of gender	HEXACO model (Ashton and Lee, 2007)	Questionnaire: HEXACO-PI-R (Lee and Ashton, 2004)	a)Students with lower openness to experience and higher extraversion show higher enjoyment and by this effort in PE b) Boys: honesty-humility shows a stronger relationship to effort via enjoyment compared to girls Girls: agreeableness shows a stronger relationship to effort via enjoyment compared to boys
Lodewyk (2018) Canada—Journal Article: *Educational Psychology*	316 students (161 m; 155 f); grade 9 and 10	Relationships between personality and(a) anxiety (b) self-efficacy, and (c) intentions to exercise as a function of gender	HEXACO model (Ashton and Lee, 2007)	Questionnaire: HEXACO-PI-R (Lee and Ashton, 2004)	Students scoring higher in extraversion show(a) lower anxiety and (b) higher self-efficacy and (c) higher intentions to exercise (f/m); Students scoring higher in openness to experience show higher anxiety (f/m) and lower self-efficacy (f)
Seitz and Bäumler (1972) Germany—Book Section	70 students (m); grade 6 (age 11–13)	Relationships between personality traits and motor performance	16PF (Cattell, 1973)	Questionnaire: CPQ (Porter and Cattell, 1963)	Students scoring higher in personality dimensions (motor activity, optimistic unconcern and distance to authority) show better results in motor performance (flexibility or movement coordination)
Westhoff (1989) Germany—Journal Article: *Sportunterricht*	31 students (15 m; 16 f); grade 7 (age 12–13)	Relationships between personality and volleyball-specific abilities	3 non-motor variables: Students' interest on PE, concept of own abilities, anxiety of social consequences	Questionnaires: assessing non-motor variables	- Students with higher volleyball-specific abilities show higher content-specific interests and higher sports-specific concept of own abilities - Weak relationship between volleyball-specific abilities and anxiety
Williams and Eston (1986) UK—Journal Article: *Physical Education Review*	30 students (m) (age Ø 16)	Relationships between personality and(a) exercise intensity and (b) perception of exertion	Two-factor model (Eysenck and Eysenck, 1969)	Questionnaire: JEPI (Eysenck, 1965)	No relationship between personality (measured via extraversion) and(a) exercise intensity or (b) perception of exertion
Willimczik (1986) Germany—Journal Article: *Sportunterricht*	73 students (37 m; 36 f); grade 8 middle school (age Ø 16)	Relationships between different internal conditions and motor learning abilities	Personality traits = cognitive psychological construct (concept of own abilities, achievement motivation, attributions, anxiety)	Questionnaires: concept of own abilities (Meyer, 1984); achievement motivation (Schmalt, 1976); attributions (Weiner and Reisenzein, 1984); anxiety	Students scoring higher in the dimension concept of personal abilities show higher learning abilities
Wilson (1969) USA—Journal Article: *Research Quarterly*	154 students; high school	Relationships between selected personality factors and motor performance	16 PF (Cattell, 1946) Temperament (Guilford, 1971)	Questionnaires: 16 PFQ (Cattell and Eber, 1962); GZTS (Guilford et al., 1949)	Negative relationship between self-reliance and motor performance
**LONGITUDINAL STUDIES**					
Bachleitner-Hofmann (1986) Germany—Series	89 students (age 14–19)	Influence of more PE on personality	Lexical trait model (Fahrenberg et al., 1978) + self-concept (Sack, 1980) + attitudes (Kenyon, 1968)	Questionnaires: FPI (Fahrenberg et al., 1978); EWL (Sack, 1975, 1980); ATPA-D (Kenyon, 1968)	T0: Sports class students score higher in sports-specific achievement orientation (attitudes) T1: Sports class students are more inhibited and reserved (traits)
Blanchard (1946) USA—Journal Article: *Research Quarterly*	164 students; grade 8–11 high school	1)Whether or not personality traits are continuous in development 2)Whether boys or girls show greater development in personality traits over a 2 year period	Personality = integrated total of traits possessed by an individual	Questionnaire: BFRS (Blanchard, 1936)	1)Continuous growth in character and personality traits with each succeeding grade level 2) Development of wholesome character and personality traits in girls is overall greater than in boys
Gabler (1976) Germany—Edited Book	254 students (age 12–13 and 15–16)	Influence of sports class participation on the development of specific personality traits	16PF (Cattell, 1973), achievement motivation as independent part of personality + interests and attitudes	Questionnaires: HSPQ (Cattell, 1973); TAT (Heckhausen, 1974)	T0: Only one significant difference between sports class students and regular class students in the dimension perfectionism T1: Dominance increased significantly in sports class students compared to regular class students
Geron (1981) Israel—Conference Proceedings	395 professional junior student athletes; junior high school (age 11–12)	Influence of sports class participation on psychological characteristics	Personality characteristics: anxiety, locus of control and reactions to frustration	Questionnaires: Trait/State Anxiety Test (Spielberger et al., 1984); TALOC (Milgram and Milgram, 1975); Picture Frustration Test (Rosenzweig, 1944)	T0: Sports class students score higher in aggression, need persistence and obstacle dominance; regular class students are characterized more conformist and ego defensive T1: Sports class students change and score higher in locus of control and reaction to frustration
Krejci (1993) Czech-Republic—Journal Article: *Social Science International*	247 students (127 m; 120 f); grade 3, 5, and 7 elementary school (age 9–13)	Psychological development of students and the possibility of forming their personality in the process of PE	Two-factor model (Eysenck and Eysenck, 1969)	Questionnaire: EPI (Krallova, 1971)	T0: No differences among the initial measurement T1: Students in the intervention group score higher in extraversion, especially boys
Mijaica (2017) Romania—Conference Proceedings	2 classes; grade 9 and 10 college (age 15–17)	Influence of a specialized curriculum on the development of personality traits	Five personality directions: leadership; managing conflicting situations; preventing conflicting situations; fair-play; sports disciplines	Systematic observation method (Epuran, 2005)	Intervention group shows a significantly higher development in terms of target skill acquisition (solving conflict situations, fair-play, leadership) compared to control group
Tillman (1965) USA—Journal Article: *Research Quarterly*	386 students; junior and senior high school	Influence of a physical fitness program on selected personality traits	16 PF (Cattell, 1946) Social behavior (Allport and Allport, 1928) Preference Record (Kuder, 1950)	Questionnaires: A.S. reaction study (Allport and Allport, 1928); 16 PFQ (Cattell and Eber, 1962)	Experimental group only differs in one personality dimension (vocational interest: clerical) compared to control group
Schubert (1973) Austria —Dissertation	185 students (f); grade 5 and 6 sports school	Influence of more PE on students' personality traits	Parts of personality: self-criticism/-control,/- confidence, initiative, contact, anxiety, satisfaction with parental home, and school	Questionnaire: SPQ (Zrzavy, 1960)	No differences between sports class students and regular class students in grade 6 Differences regarding satisfaction in school in grade 5: sports class students are more satisfied in general school than non-sports class students
Zupancic and Justin (1998) Slovenia—Journal Article: *Educational Research and Evaluation*	62 professional junior student athletes; grade 2 grammar school (age 16–17)	Impact of sports classes on personality development	16PF (Cattell, 1973)	Questionnaires: polish version of 16 PFQ (Lamovec, 1975); profile index of emotion (Plutchik and Kellerman, 1974)	T0: High performing sports class students are more achievement oriented, have a stronger ego, behave more spontaneously, are less demanding and less depressed than regular class students T1: High performing sports class students undergo more changes in personality traits compared to regular class students—increased dominance, ego strength, surgency, sophistication or decreased anxiety, and depressed moods in high performing sports class students

## Results

Figure 1 summarizes the search and review process, differentiating between the initial and the update search. Both searches in total yielded 3,963 references. After removing duplicates, screening titles and abstracts 91 full-texts were examined. Twenty-three references fulfilled all inclusion criteria and were therefore considered for analysis. One additional reference was included via the reference list search. The author and key journal search did not yield any additional reference. In total, 24 references were included in the review.

### Framework Conditions of the Included Studies

Most of the studies (*n* = 18) were implemented in Europe, eight thereof in Germany (Seitz and Bäumler, 1972; Dunkerbeck and Prenner, 1976; Gabler, 1976; Friedrich, 1978; Bachleitner-Hofmann, 1986; Willimczik, 1986; Westhoff, 1989; Klein, 2017) and three in the United Kingdom (Kerr, 1978; Williams and Eston, 1986; Hayes, 2017). Five studies originated from Canada or the United States of America (Blanchard, 1946; Tillman, 1965; Wilson, 1969; Lodewyk, 2018; Lodewyk and Gao, 2018). The remaining studies originated from Austria, Croatia, Czech Republic, Israel, Poland, Romania and Slovenia. The included studies were published between 1946 and 2018, inclusive. Nine studies thereof were conducted before 1980, seven between 1980 and 2000 and eight after 2000. Seventeen studies were published in 14 different journals (10 thereof peer-reviewed). Four studies were published as books or chapters in an edited book, two studies were published within conference proceedings and one study was a dissertation. Dunkerbeck and Prenner (1976) asked 50 PE teachers to describe their students' personality. In the remaining 23 studies participants were students between 5 and 19 years. Most of the studies investigated teenaged students between 14 and 17 years. The number of participants in all studies ranged from 30 to 1,399. Eight studies observed <100 participants, 14 studies examined between 100 and 600 participants and two studies recruited more than 1,000 participating students. Fifteen of the included studies were cross-sectional studies, nine longitudinal. Longitudinal studies lasted from 6 months to 5 years.

### Personality Understanding of the Included Studies

The studies followed different understandings of personality. Most of the studies (*n* = 17) followed trait theory and either applied the 16 PF model of Cattell (1946) (*n* = 8), the two- or three-factor model of Eysenck (1981) (*n* = 4), or the five-factor model of McCrae and Costa (1992) (*n* = 5). Four studies (Tillman, 1965; Wilson, 1969; Kerr, 1978; Guszkowska and Rychta, 2007) united different personality approaches in their research. Others (*n* = 8) understood personality as an interaction of several factors, such as self-feelings, feelings toward others, anxiety, locus of control, or reactions to frustration (Blanchard, 1946; Schubert, 1973; Dunkerbeck and Prenner, 1976; Geron, 1981; Willimczik, 1986; Westhoff, 1989; Hayes, 2017;Mijaica, 2017).

The included studies used different methods to operationalize personality. The majority (*n* = 21) used questionnaires to assess quantitative data and applied 19 different inventories. One study (Mijaica, 2017) used assessment sheets (Epuran, 2005) in order to systematically observe specific behavior indicating students' personality traits. Two studies (Dunkerbeck and Prenner, 1976; Hayes, 2017) applied a semi-structured interview or free descriptions to capture qualitative information.

### Research Questions and Results of the Included Studies

The studies can be classified into three thematically coherent groups: Two groups depict cross-sectional studies and one group unites all longitudinal studies. One group of cross-sectional studies focused on the relationships between students' personality and their achievement in PE. The remaining cross-sectional studies examined relationships between students' personality and students' psychological determinants of PE participation e.g., motivation in PE or attitudes toward PE. All of the longitudinal studies investigated the influence of a school sports intervention on students' personality or rather their personality development.

#### Relationships Between Students' Personality Traits and Achievement in PE

Ten of the cross-sectional studies (Wilson, 1969; Seitz and Bäumler, 1972; Dunkerbeck and Prenner, 1976; Friedrich, 1978; Kerr, 1978; Williams and Eston, 1986; Willimczik, 1986; Westhoff, 1989; Guszkowska and Rychta, 2007; Culjak and Mlačić, 2014) focused on the relationships between students' personality traits and their achievement in PE.

Two studies examined the relationship between students' personality traits and their PE grade (Friedrich, 1978; Culjak and Mlačić, 2014). Culjak and Mlačić (2014) showed relationships between Goldberg's conscientiousness, extraversion and emotional stability and better grades and therefore success in PE. These relationships were different for male and female students. Male students' (16–17 years) success was positively related to conscientiousness and negatively to extraversion. Female students' (16–17 years) success was positively related to conscientiousness and emotional stability. In Friedrich's (1978) study, extraverted students (12 years) achieved better grades in PE.

Six studies (Wilson, 1969; Seitz and Bäumler, 1972; Dunkerbeck and Prenner, 1976; Kerr, 1978; Williams and Eston, 1986; Guszkowska and Rychta, 2007) analyzed the relationship between students' personality traits and their motor performance in PE. All studies except one (Williams and Eston, 1986) described a clear relationship between personality traits and different aspects of motor performance. Kerr (1978) showed that ball skills performance was positively related to Cattell's (1946) personality characteristics warmth, emotional stability, dominance, liveliness and Eysenck's dimension extraversion, but negatively related to Cattell's (1946) sensitivity, social boldness and apprehension as well as to Eysenck's (1981) introversion. Wilson (1969) found a negative correlation between Cattell's (1946) self-reliance and motor performance. Students (11–13 years) scoring higher in Seitz and Bäumler's (1972) personality dimensions motor activity, optimistic unconcern and distance to authority showed better results in flexibility and movement coordination (Seitz and Bäumler, 1972). Dunkerbeck and Prenner (1976) showed differences between high performing and low performing students regarding their personality and behavior assessed by the PE teacher. According to the interviewed PE teachers, low-performing students were shyer, more timid, and less social (Dunkerbeck and Prenner, 1976). Boys (15–17 years) in Guszkowska and Rychta's (2007) study obtained a greater number of significant correlations between personality and motor performance than peer girls. Extraversion e.g., was positively correlated with boys' total fitness score. In addition, agreeableness was positively correlated with agility, trunk muscle strength and suppleness; trunk muscle strength and suppleness also with conscientiousness. None of these correlations were found for girls. Williams and Eston (1986) did not detect any relationship between personality—measured only via extraversion—and fitness or effort perception.

The remaining two studies (Willimczik, 1986; Westhoff, 1989) in this group focused on the relationship between students' personality traits and motor learning abilities. Both studies described the concept of personal abilities within their personality understanding and showed a positive relationship to higher learning abilities (12–13 years; 16 years, respectively). Apart from that, the studies revealed only few significant results. The relationship between interest in PE and student performance in PE e.g., was significant for boys and girls. Anxiety about social consequences was more prominent in girls and negatively related to their motor learning abilities (Westhoff, 1989). Boys scoring higher in hope for success performed better. Girls performed better when scoring lower in fear of failure (Willimczik, 1986).

#### Relationships Between Students' Personality and Their Psychological Determinants of PE Participation

All five cross-sectional studies in this group (Erpic et al., 2005; Hayes, 2017; Klein, 2017; Lodewyk, 2018; Lodewyk and Gao, 2018) investigated and detected relationships between students' personality traits and several psychological determinants of PE participation. Erpic et al. (2005) examined the relationships between students' personality traits and their motivation in and attitudes toward PE. Students (11–18 years) scoring higher in conscientiousness show more positive attitudes toward PE. Students achieving higher scores in neuroticism and lower scores in agreeableness are less motivated in PE. Erpic et al. (2005) concluded that personality traits are related to students' motivation to learn and to perform in PE classes. Klein (2017) analyzed the relationship between general personality traits and physical self-concept. A high score in neuroticism was related to a lower assessment of physical attractiveness and athleticism. A weaker but positive correlation was shown between extraversion and athleticism. Hayes (2017) investigated the development of negative attitudes toward PE with the aid of a semi-structured interview and identified personality as one developmental factor. Due to the fact that the impact of students' personality traits on their enjoyment and engagement in PE is difficult to assess, Hayes (2017) suggested to consider personality-related predictors of PE enjoyment and engagement instead e.g., resilience, intrinsic motivation, and confidence. Lodewyk and Gao (2018) focused on the relationships between students' (14–15 years) personality traits and various outcomes such as enjoyment and effort in PE. By means of a proposed model, they showed that lower openness to experience and higher extraversion are related to a higher level of enjoyment. Further a higher level of enjoyment is related to a higher level of effort. In a second study, Lodewyk (2018) investigated the relationships between personality traits and anxiety, self-efficacy and intensions to exercise. This study showed that higher extraversion is associated with lower anxiety, higher self-efficacy, and a higher level of intentions to exercise in both males and females (14–15 years). Furthermore, higher openness to experience is associated with raised anxiety and lowered self-efficacy in females.

#### Influence of a School Sports Intervention on Personality

Five of the longitudinal studies analyzed personality differences between students participating in sports classes (receiving a higher amount of PE per week) and students participating in regular classes. Sports class students in Schubert's study (Schubert, 1973) received four additional PE lessons per week. The remaining four studies did not specify the amount of additional PE. In two of the studies, students of sports classes were professional junior athletes (Geron, 1981; Zupancic and Justin, 1998). These studies aimed at identifying potential personality differences between high performing student athletes and regular class students (t0 and t1) as well as at examining their personality development (t1). Zupancic and Justin (1998) showed that sports class students (16–17 years) were more natural, spontaneous and undemanding whereas regular class students were more propulsive and intellectual with a self-interested attitude in the initial measurement. In addition, sports class students were more practically oriented, conformist and more worried about everyday necessities, but able to stay calmer in crucial situations (autia-praxernia). Furthermore, sports class students were more controlled over emotions, showed more discipline and a higher self-esteem (integration) (Zupancic and Justin, 1998). Geron (1981) showed initial personality differences in the dimension of reaction to frustration. Sports class students (11–12 years) scored higher in aggression, need persistence and obstacle dominance whereas regular class students were characterized as more conformist and ego defensive (Geron, 1981). Furthermore, Geron (1981) highlighted initial differences between sports class students and regular class students regarding their personality structure. Compared to regular class students, sports class students' motor skills and behavioral characteristics depended less on their socio-economic status. Comparing data of the first and second measurement point within the groups, both studies emphasized that sports class students' personality traits changed more or rather developed into contradictory directions compared to regular class students. In Zupancic and Justin's study (Zupancic and Justin, 1998), sports class students dropped on the deprivation and on the anxiety scale, whereas in the regular class group the mean score for deprivation increased over the 2 years. The initial differences between the two groups regarding the dimensions autia-praxernia and integration were no longer significant. In addition, sports class students increased their score in their ego strength, dominance, surgency as well as their score in sophistication. Regular class students obtained an insignificant increase in the same dimensions (Zupancic and Justin, 1998). Geron (1981) concluded that sports class students had changed in the dimensions locus of control and reaction to frustration after 1 year. A positive development was highlighted for sticking to rules, working within a framework, self-control, and perseverance. The authors did not report a change among the regular class students.

Three studies investigated sports class students who signed up for sports classes but were not professional athletes (Schubert, 1973; Gabler, 1976; Bachleitner-Hofmann, 1986). Sports class students (14–19 years) in Bachleitner-Hofmann's (1986) study scored higher in sports-specific achievement orientation which he declared as part of their personality. In two studies, the first data assessment took place after a 1 year participation in the sports class (Schubert, 1973; Gabler, 1976). Schubert (1973) did not detect personality differences between the intervention and the control group enrolled in regular classes. Gabler (1976) only found one significant difference in the dimension perfectionism. Thus, students in sports classes are less concerned and show less self-discipline regarding social norms than students in regular classes. Comparing the two groups at the second measurement point, in Gabler's study (Gabler, 1976) differences in perfectionism were still present. Changes between the first and second assessment were similar for both groups, except for dominance, which increased significantly in sports class students but not in regular class students. The other two studies also detected only few significant differences with e.g., sports class students being more inhibited and reserved (Bachleitner-Hofmann, 1986) and more satisfied in school (Schubert, 1973) than regular class students.

The nine longitudinal studies investigated the influence of a school sports intervention on students' personality, either through specific sports programs (different didactical alignment and structuring of PE lessons) or by participation in sports classes (receiving a higher amount of PE per week). Four studies (Blanchard, 1946; Tillman, 1965; Krejci, 1993; Mijaica, 2017) focused on the influence of specific PE programs on students' personality or personality development. One study (Blanchard, 1946) did not consider a control group. Blanchard (1946) investigated boys and girls (grade 8–11) from PE classes and analyzed differences between the sexes. During the intervention, students experienced various sports (boys: football, basketball, gym classes; girls: basketball, volleyball, shuffleboard, soft ball, gym classes). This study detected the greatest gain over time in the dimensions ethical social qualities (truthful, fair) and qualities of efficiency (dependable, trustworthy). Overall, gains in girls were greater than in boys. Tillman (1965), Krejci (1993), and Mijaica (2017) examined the impact of a specific PE program (intervention group) on students' personality traits compared to regular PE (control group). Krejci (1993) and Mijaica (2017) detected changes in personality traits within the intervention group and in comparison to the control group. Students (9–13 years) in Krejci's (1993) intervention group experienced PE lessons that emphasized social learning by implementing special games or adapting PE teacher behavior. After the intervention, students in the intervention group scored higher in extraversion, especially boys and depicted more positive attitudes toward PE (Krejci, 1993). Students (15–17 years) in the intervention group—experiencing personality development supportive units characterized by an array of games, targeting at educational objectives, values and attitudes—showed a significantly greater development of targeted skills (e.g., leadership, problem-solving, fair-play), typifying personality development (Mijaica, 2017). Tillman (1965) followed a special study design with a first study phase in which male junior and senior high school students were classified into two groups based on their results in a physical fitness test (lower 15% vs. upper 15%). Between these groups he found significant personality differences (upper 15% more dominant, extraverted and socially oriented). In a second study phase he divided the lower 15 percent in an intervention and a control group, with the intervention group receiving 9 months strenuous physical fitness training instead of regular PE. After the intervention, the intervention group scored significantly higher in physical fitness but only in one (clerical interest) out of 28 personality dimensions.

## Discussion

The aim of the review was to give an overview of the literature dealing with students' personality in PE. The underlying personality understandings of the included studies are inconsistent in general. More recent studies though exhibit greater consistency. The research field investigates relationships between students' personality and (a) students' achievement in PE or between students' personality and (b) their psychological determinants of PE participation or (c) the influence of a school sports intervention on students' personality. Relationships regarding personality were found in all three groups—(a), (b), and (c). The following discussion is divided into two parts: (1) Discussion of personality understandings and (2) Discussion of research questions and results—separately for (a), (b), and (c).

### Discussion of Personality Understandings of the Included Studies

Among the included studies, three models are predominant to approximate the understanding of personality: The models of Cattell (1946); Eysenck (1981); McCrae and Costa (1987). The fact that all three models follow personality's trait approach (John et al., 2008), signalizes this approach as the leading paradigm in students' personality research in PE. Following the trait approach is very common in general personality research (Novikova, 2013) as well. Using trait psychological models in the educational context is less common—because of the focus on learning theories—but nonetheless existent in educational studies. O'Connor and Paunonen (2007) and Poropat (2009) in their studies for example made use of the trait approach in order to analyze relationships between students' personality traits and their academic performance.

Most of the elder studies (1946–1986) (Tillman, 1965; Wilson, 1969; Seitz and Bäumler, 1972; Gabler, 1976; Kerr, 1978; Bachleitner-Hofmann, 1986) follow the 16PF model of Cattell (1946). Studies between 1978 and 1993 (Friedrich, 1978; Kerr, 1978; Williams and Eston, 1986; Krejci, 1993) primarily use Eysenck's (1981) model of personality. Using the five-factor model (McCrae and Costa, 1987) or its further development, e.g., the HEXACO-model (Honesty-humility, Emotionality, eXtraversion, Agreeableness, Conscientiousness, Openness to experience) (Lee and Ashton, 2004), is more frequent in recent studies (2005–2018) (Erpic et al., 2005; Guszkowska and Rychta, 2007; Culjak and Mlačić, 2014; Klein, 2017; Lodewyk, 2018; Lodewyk and Gao, 2018). This is in line with the five-factor model's dominance in contemporary psychology in the last two decades (McCrae, 2001; Rammstedt et al., 2012). The abovementioned trajectory can also be retrieved in general personality research, beginning with Cattell's model, followed by Eysenck's model to McCrae and Costa's five-factor model of personality. Considering the included studies in our review, all three models—Cattell (1946); Eysenck (1981); McCrae and Costa (1987)—are deployed in each of the three groups with the five-factor model being primarily used in studies investigating students' personality in relation to their psychological determinants of PE participation. Although the three models are predominant in the reviewed studies, some of the researchers created or assorted and by this examined their own understanding of personality (Blanchard, 1946; Schubert, 1973; Dunkerbeck and Prenner, 1976; Geron, 1981; Willimczik, 1986; Westhoff, 1989; Hayes, 2017; Mijaica, 2017). This holds true even in recent studies where the trait approach had become dominant and widely accepted. Even if the trait approach is generally accepted, the results of our review signify that in addition to following the trait approach, other facets of personality are implied in PE research. Several researchers expand their underlying understanding of personality by examining other person-related facets such as self, interests or achievement motivation (Gabler, 1976; Bachleitner-Hofmann, 1986; Willimczik, 1986; Erpic et al., 2005).

In our review, all studies following the trait approach use questionnaires to measure personality. Questionnaires therefore can be seen as methodology of choice when operationalizing personality within a clear underlying personality understanding. It is remarkable that even if the majority of the included studies follow the trait approach, 19 different inventories are used to measure personality. A possible reason might be that during the trait approach's development many different inventories were created and used in personality research relative to the respective research aim or sample under investigation. Comparing results of studies that apply similar inventories, is—due to the similar development and background of the models—possible, but requires a careful and often time-consuming comparative analysis. Three of the included studies (Dunkerbeck and Prenner, 1976; Hayes, 2017; Mijaica, 2017) collected qualitative data and applied their own understandings of personality instead of following an established personality approach. Therefore, these results are only content-wise comparable among themselves or to other studies in the review.

### Discussion of Research Questions and Results of the Included Studies

#### Studies Investigating the Relationships Between Students' Personality Traits and Achievement

Nine out of ten studies found relationships between students' personality and their achievement in PE. Similar findings could also be retrieved in other settings, e.g., in competitive sports. In their review Allen and Laborde (2014) e.g., analyzed contemporary studies to find evidences for personality traits as precursors to athletic success in terms of sports performance. They concluded that athletic success in competition and participation in physical activity could be predicted by personality traits (Allen and Laborde, 2014). Studies investigating the relationships between students' personality traits and achievement in PE operationalized achievement differently. This fact had to be considered while discussing the studies' results.

Studies in our review revealed that extraversion is notably related to students' PE grade. The direction of the relationship is diverse though among the studies: Friedrich (1978) detects a positive relationship whereas Culjak and Mlačić (2014) indicate a negative relationship. This might be caused by the long period of time between the studies and the concomitant change in the education system as well as by the different cultures in which the two studies were conducted. Furthermore, there is no uniform picture regarding grading practice, which might explain why each study consults different criteria to compose students' PE grade. In order to find out whether extraversion has a positive or negative influence on students' PE grade, the grade's composition needs to be determined and other influencing factors (such as the teacher or the students' performance) must be monitored. Similar to Culjak and Mlačić's (2014) detected positive relationships between girls' emotional stability and their PE grade, Steca et al. (2018) showed that successful athletes obtain higher emotional stability than less successful athletes. Additionally, conscientiousness is in the included studies of our review positively related to students' PE grade, which is in line with general educational research where conscientiousness is considered a crucial non-cognitive determinant of school grades (Dumfart and Neubauer, 2016).

Students' performance measured by fitness or ability tests is also positively related to extraversion - independent of the chosen methodology: Either when measured by Cattell's warmth and liveliness (Kerr, 1978), Cattell's self-reliance (Wilson, 1969), Eysenck's extraversion (Kerr, 1978) or as highlighted in Seitz and Bäumler's (1972) and Dunkerbeck and Prenner's (1976) findings. Similar findings are known from research considering leisure time physical activity or competitive sports (Shariati and Bakhtiari, 2011). Shariati and Bakhtiari (2011) indicate that athletes scored higher in extraversion than non-athletes. This is in line with research showing that more extraverted individuals are also more energetic (Terracciano et al., 2013) which is also supported by findings that extraverted individuals tend to exercise more in their free time and therefore probably perform better (Rhodes and Smith, 2006). These explanations emphasize selection processes in sports whereas the assumption that sports promote extraversion supports the impact of socialization processes. According to Gerlach (2008), it can be assumed that selection processes first pave the way for sports or physical activity, in which adolescents then experience a corresponding socialization.

Besides extraversion, Dunkerbeck and Prenner (1976) as well as Guszkowska and Rychta (2007) report relationships between performance and conscientiousness by measuring conscientiousness directly or describing high performing students as hard-working and ambitious—characteristics that accompany conscientiousness (McCrae and Costa, 1987). In general educational research, out of all five personality dimensions conscientiousness is most strongly and consistently associated with academic performance. This dominant relationship cannot be found when considering PE specifically. A possible explanation might be that other subjects are more closely linked to academic performance than PE: The PE grade consists of e.g., motoric, social, cognitive components, whereas other subjects' grades depict usually a purely cognitive achievement (Roth et al., 2015). Kerr (1978) with his results on Cattell's dimensions emotional stability and apprehension shows that neuroticism is negatively associated with students' performance in PE. Guszkowska and Rychta's (2007) results support this relationship for boys. Same is known for successful athletes showing higher emotional stability than less successful athletes or non-athletes (Steca et al., 2018). Accordingly, emotional stability benefits good performance in various contexts, not only in school PE.

In summary, relationships between students' achievement in PE and their personality are partly comparable to results of studies in leisure sports or general educational research. Considering extraversion and conscientiousness however, contradictory relationships became apparent. This fact underlines PE's above-mentioned specific demands regarding students' performance in comparison to other school subjects.

#### Studies Investigating the Relationships Between Students' Personality and Their Psychological Determinants of PE Participation

Due to the fact that the students' psychological determinants of PE participation differ among the analyzed studies, the highlighted relationships are barely comparable. Considering the different determinants—motivation (Erpic et al., 2005), self-concept (Klein, 2017), attitudes to PE (Hayes, 2017), enjoyment (Lodewyk and Gao, 2018), anxiety, self-efficacy, and intentions to exercise (Lodewyk, 2018)—findings from general educational research are similar: Students' personality—commonly measured by inventories based on personality's trait approach, similar to the studies in our review—is related to students' academic (intrinsic) motivation (Komarraju et al., 2009), self-concept (Pilarska, 2018), attitudes toward school (Heaven et al., 2002), enjoyment in life (Cheng and Furnham, 2003) as well as test-anxiety (Chamorro-Premuzic et al., 2008), self-efficacy (Caprara et al., 2011), and exercise intentions (Rhodes et al., 2003). The fact that the relationships detected in PE context coincide with relationships detected in general educational context underlines personality's important role in education.

In the analyzed studies extraversion is positively related to a positive physical self-concept (Klein, 2017), a high score in PE enjoyment (Lodewyk, 2018), high self-efficacy, positive intentions to exercise and low anxiety (Lodewyk, 2018). Similar relationships were found in the general educational context for extraversion and general self-esteem (Pilarska, 2018), life enjoyment (Cheng and Furnham, 2003), and high intentions to exercise (Rhodes et al., 2003). One explanation for the strong relationships in PE context shown in our review might be that PE demands social interaction and cooperation more than other subjects. Extraverted students feel more comfortable because they are more sociable and seek the company of others. This is in line with the aforementioned assumption that extraverted people are more physically active (Rhodes and Smith, 2006), perform better and therefore possibly feel more comfortable when exercising. However, the question that remains unanswered is whether these findings are actually PE specific or whether they are attributable to and domain-specific for sporting activities in general.

Regarding conscientiousness, the analyzed studies in our review only report relationships with positive attitudes toward PE whereas studies in other subjects emphasize conscientiousness as strong predictor of further inner facets such as motivation, self-efficacy, self-control and self-esteem (e.g., Heaven et al., 2002; Komarraju et al., 2009; Caprara et al., 2011; Pilarska, 2018). A possible explanation for the diminished relationship with conscientiousness might be the weak link between PE and academic performance. In other subjects, variables such as motivation or self-efficacy act as mediators within the strong relationship between conscientiousness and academic performance. Compared to other school subjects, academic performance's role is less significant in PE (Roth et al., 2015). This might be reason for the weaker relationship between conscientiousness and e.g., motivation, self-concept and enjoyment in PE.

In addition, our review shows interesting relationships between students' psychological determinants of PE participation and openness to experience which is negatively related to enjoyment (Lodewyk and Gao, 2018) and self-efficacy and positively related to anxiety (Lodewyk, 2018). Contradictory to the studies' results of our review (Erpic et al., 2005; Lodewyk, 2018; Lodewyk and Gao, 2018), openness to experience is in other contexts positively related to learning motivation (Hazrati-Viari et al., 2012; Wahyu Ariani, 2013), positively associated with enjoyment (Lindenberg, 2001), positively related to academic self-efficacy (Sánchez-Cardona et al., 2012), and unrelated to anxiety (Kotov et al., 2010).

These contrary results again underline the fact that PE compared to other subjects demands different student abilities. In PE the demanded abilities are less associated with intellectual performance e.g., PE teachers still often use teacher-centered instructional styles (Byra, 2006; Pfitzner, 2014), which go along with a clear and predetermined lesson structure. Further, PE often focusses on student performance (Rink, 2013) and therefore does not necessarily address openness to experience. People scoring high in openness to experience are described as aesthetic appreciating, inquisitive, creative and unconventional (Lee and Ashton, 2004). They enjoy to educate themselves in the intellectual, artistic and historical fields—closely associated with learning environments (Moshagen et al., 2014). This could explain why openness shows different relationships in other school contexts, e.g., students who are intellectually curious are more likely to enjoy learning (Tempelaar et al., 2007; Komarraju et al., 2009). PE in contradiction might be rather unpopular for students who score high in openness and are therefore more inclined toward learning situations. A new teaching style and alternative forms of teaching—e.g., experiential learning, genetic learning or generally student-centered, inductive and participatory teaching—might produce different results.

To summarize, the analyzed studies in our review describe several relationships between students' general personality traits and psychological determinants of PE participation. The findings in our review compared to findings in general educational research emphasize PE's unique role in the curriculum—being the only subject demanding and developing cognitive, social as well as physical competencies. PE challenges different needs whereby determinants such as physical self-worth or anxiety become important.

#### Studies Investigating the Influence of a School Sports Intervention on Students' Personality

Interesting and discussable are the differences between high performing student athletes in sports classes and regular class students e.g., regarding Cattell's dimensions autia-praxernia and integration (Geron, 1981; Zupancic and Justin, 1998), which are mainly associated with conscientiousness: High performing student athletes score higher in conscientiousness (Zupancic and Justin, 1998). Studies in other contexts detect similar relationships. Athletes or physically active people score higher in the dimension conscientiousness (Rhodes and Smith, 2006; Malinauskas et al., 2014). Results differ regarding the level of professionalism: Athletes competing at a higher level score higher in conscientiousness than athletes competing at a lower level (Allen et al., 2011). Self-discipline and organization are prerequisites of a physically active lifestyle (Rhodes and Smith, 2006; Gallagher et al., 2013) encouraging conscientiousness, which possibly explains the abovementioned finding. However, the reviewed studies do not answer the question whether high performing athletes differ because of the sports they practice or due to the fact that they are generally different. The effect of selection processes as well as socialization processes seems to occur, as was shown in studies considering students' self-concept (Brettschneider, 2002; Stiller and Alfermann, 2005; Gerlach, 2008). The development process of high performing student athletes and regular class students also differs, which in turn may indicate that sports influences personality development. It remains unclear though, whether different processes of personality development are caused by sports class enrollment merely or probably more likely by performing competitive sports. The assumption that competitive sports may have a significant influence is supported by studies investigating the influence of competitive sports on adolescents' personality development (Conzelmann, 2001) as well as by the fact that studies in our review which examine sports classes but not high performing athletes reveal only minor differences in terms of personality development. Students interested in sports or practicing more sports do not seem to be different *per se* or differ considerably in their personality development. However, the personality of students in sports classes considering high performing student athletes develops differently.

This result is also detectable in studies examining special PE programs. It has to be mentioned though that observed changes are rare and only detected by individual studies. Regarding extraversion, Krejci (1993) found an increase of extraversion in the intervention group similar to the results in high performing student athletes (Zupancic and Justin, 1998). Similar results were also found for general physical activity, where extraversion was identified as determinant of physical activity (Rhodes and Smith, 2006). Reasons for the higher scores can be the necessity to cooperate with others or to assert oneself in competition—both typical situations in PE. Zupancic and Justin (1998) assumed that sports class students undergo more extensive life experiences through training and competing in various environments and thus extraversion is promoted. In addition, Costa et al. (2005) and Pocnet et al. (2013) declared biological and cognitive processes responsible for increased extraversion in physically active people. Physical activity can reduce e.g., disease burden, cognitive decline, and risk of depression associated with low scores in extraversion (Costa et al., 2005; Pocnet et al., 2013). Contrary to increased conscientiousness in high performing athletes (Zupancic and Justin, 1998), the other reviewed studies do not show an increase in conscientiousness. Gabler (1976) even highlighted a decrease in Cattell's (1956) dimensions perfectionism and rule-consciousness associated with the second-order factor self-control which complies with conscientiousness (Rossier et al., 2004). According to this, sports class students are less conscientious than regular class students. This insight again supports the assumption that competitive sports may be decisive for personality development, possibly due to the concomitant participation in competitions and athletes' high motivation and willingness to perform. The missing relationship regarding non-high performing sports class students might be caused by PE's contextual peculiarity, as physical activity is part of the school curriculum and thus compulsory for students. Unlike professional athletes, students do not need to motivate themselves to be physically active and discipline themselves to be successful. This might be a reason why the analyzed intervention studies do not reveal an increased score for conscientiousness.

The results show that PE can only to a certain extend influence students' personality. This result is legitimate, as PE rather aims at supporting students' personality development than changing personalities. The assumption is supported by Tillman (1965) study in our review, where a 9-month fitness training program led to almost no changes in personality traits. In addition, the association seems to depend on many factors, including e.g., PE's curriculum or structuring. According to the studies in our review, which report hardly any changes (Tillman, 1965; Schubert, 1973), it can be assumed that PE's pedagogical alignment has a greater impact on personality development than physical activity itself. In order to test whether and to what extent PE can support personality development, it is necessary to implement a specifically designed intervention.

#### Relevance of Personality Research in PE

The findings of our review indicate that personality research can be used to teach PE in a student-centered way and by this support students' uptake of leisure time physical activity and the development of an active lifestyle—one of PE's two main goals (“educating to sports”). In order to achieve *education to sports*, PE teachers need to know students' motives to be physically active and teach PE in a varied, multi-perspective way. In view of the fact that certain general personality traits are also related to various psychological determinants of PE participation, knowing students' personality can help teachers to align PE lessons to students' needs. Our review e.g., reveals a negative relationship between neuroticism and motivation in PE (Erpic et al., 2005) and between neuroticism and PE grade (Culjak and Mlačić, 2014). Girls scoring low in neuroticism e.g., receive better grades in PE than girls scoring high in neuroticism. People scoring high in neuroticism generally are more fraught, anxious, worried, concerned, nervous, plaintive, and with self-doubt (Ostendorf and Angleitner, 2004). All these characteristics are rather unfavorable for enjoying a great number of typical PE situations where a determined task has to be fulfilled, often in new and insecurely experienced settings. Therefore, in order to engage emotionally instable students in PE e.g., the teacher has to provide tasks that satisfy the students' personality traits. The PE teacher e.g., can apply open forms of learning where students can participate in lesson decisions and freely choose from a variety of learning materials and activities. By this, the students try themselves out in activities they feel comfortable with doing and/or control their own working pace even in less secure situations avoiding the emergence of anxiety and insecurity. Further, reflecting on what has been learned, taking into account one's own emotional state, can contribute to making an initially uncomfortable task profitable and fearless in the future. The assessment of one's own level of proficiency and the subsequent personal objectives allow for an individual orientation and encourage the learner to achieve realistic and satisfactory goals. This orientation promotes the students' autonomy and competence experience and by this contributes to the satisfaction of their basic needs, which can increase their motivation (Ryan and Deci, 2017). Furthermore, attention to individual learning progress can reduce students' experience of stress and thus anxiety. This is in line with the recommendation to apply self-referenced grading in addition to criterion-referenced grading when assessing students' performance in PE (Jaitner, 2013). Considering students' personality already in lesson preparation is in line with widespread planning models for PE. Döhring and Gissel (2016) e.g., attribute students' prerequisites a crucial role in the teacher's planning of PE lessons. Students' needs and personalities have to be considered in order to carry out PE lessons as smooth and individual as possible and by this ideally reach all students.

With regard to PE's second main goal (“educating through sports”), findings of our review indicate that PE contributes to students' personality development. Several of the analyzed studies (Blanchard, 1946; Geron, 1981; Mijaica, 2017) concluded that personality traits are affected and primarily desirable traits are stimulated by participating in PE classes. However, the interventions' effects are rather small, which seems to be evident considering that PE represents only a fraction of children's everyday lives and considering that non-cognitive personality traits—examined in the analyzed studies—are relatively stable. Even in the studies with younger participants, where a less stable personality is assumed (Neyer and Asendorpf, 2018), only limited changes can be observed. Considering students' age in general, no discussable trends can be depicted in the included studies. This might be due to the studies' diverse methodologies and research aims though. Examining personality facets with a higher variability, e.g., facets of the self (Shavelson et al., 1976; Gore and Cross, 2011), is probably more suitable in intervention studies. These studies though were not included in our review, as they did not explicitly claim to assess personality. Variable personality facets, e.g., hierarchal lower-order self-concept facets should be considered in didactic concepts specifically addressing students' personality development. As a result, PE must follow targeted and pedagogically oriented concepts in order to develop students' personality and by this achieve its main goals.

## Conclusion

Our scoping review showed that research on the students' personality in PE exists, but the studies' underlying personality understandings, research questions and results are diverse. Due to the fact that the term personality was approached very broadly and we explicitly searched for this term, only studies that actually contain the term were included. Studies investigating single facets of personality without claiming to assess personality were therefore excluded. Literature reviews including several terms related to personality could provide information about further interesting relationships. Moreover, it has to be mentioned that due to feasibility reasons only German and English studies were included. Including studies published in further languages, could possibly increase the final number of included studies and provide insights into further international findings.

In addition to the aforementioned short section on ideas for further research resulting from our review's limitations, the following section will make use of the review's results and associated strengths to provide concrete practical ideas and further research opportunities. In order to explicitly highlight teaching opportunities and support PE teachers, ideas to make use of the students' personality, explicitly address students' personality or determine the specific influence of PE on students' personality development, further studies are needed:

1. Even though only studies proclaiming to assess personality were included, promising relationships between individual personality facets (e.g., interests) and learning outcomes (e.g., performance in PE) became visible when the examined facets were part of the studies' personality understanding. Therefore, a closer look at the relationships between further personality facets (e.g., self-concept, motives) and other desirable outcomes of PE (e.g., motivation, enjoyment, and achievement) would be desirable. Due to the fact, that 16 of the included 24 studies are more than 20 years old and therefore older than the existence of the nowadays widely accepted five-factor model, they display a rather inconsistent understanding of personality. For future research, high-quality studies following a clearly defined understanding of personality and applying reliable inventories should be carried out. This allows to compare results and by this receive empirical evidence.
2. It would also be interesting, to further examine the relationships between students' personality and their motives to be physically active. This knowledge allows to provide specific recommendations for PE in general and PE teaching specifically. Knowing e.g., if extraverted students are more competition- or fitness-oriented can help PE teachers to plan and structure their lessons but also to adapt their behavior when teaching in order to reach the students' diverse motives to be physically active and by this motivate them for PE in the short term and ideally for a physically active lifestyle in the long term.
3. However, it is not realistic that a PE teacher knows and considers the personality or the motives of each individual student. Further research is therefore needed to identify compositions of personality traits that are particularly important for PE enjoyment and achievement. A suitable way to further reduce complexity could be to identify typical personality patterns. Considering specific groups or types of students in PE rather than considering each individual may therefore facilitate PE planning and teaching. Müller et al. (2013) and Burrmann (2015) have already implemented similar approaches. The authors identified typical sub-groups that differ in their self-concept or in their perception of PE, respectively. Burrmann (2015) concluded that further research regarding students' personality types could be beneficial in order to realize student-centered teaching and by this promote PE's two main goals—“educating to sports” and “educating through sports.”
4. Besides intensifying research that addresses students' personality by explicit and adapted teaching, interventions aiming at students' personality development raise hope for future research. It seems to be promising to target interventions at specific and individual personality facets (e.g., anxiety, self-confidence). The more the interventions' content corresponds to the examined facets, the more likely the intervention influences the facets under examination and by this the students' personality (Conzelmann et al., 2011). Teaching methods explicitly promoting students' personal development such as problem-based learning or experiential learning already exist and might be worth considering and utilizing in such targeted interventions.

By providing the abovementioned practical opportunities but also further research ideas for PE, we aimed at deepening and specifying the results of our review in order to increase the chances of achieving PE's main goals in the long term.

## Author Contributions

AK, MS, FM, and SS conceived and designed the study. AK and MS performed the literature search and study selection process. AK, MS, and SS performed the final analysis process. AK wrote the paper with substantial contributions from MS as well as SS and FM. All authors approved the final version of the manuscript.

### Conflict of Interest

The authors declare that the research was conducted in the absence of any commercial or financial relationships that could be construed as a potential conflict of interest.
